# Molecular mechanism of parallel fiber-Purkinje cell synapse formation

**DOI:** 10.3389/fncir.2012.00090

**Published:** 2012-11-23

**Authors:** Masayoshi Mishina, Takeshi Uemura, Misato Yasumura, Tomoyuki Yoshida

**Affiliations:** ^1^Brain Science Laboratory, The Research Organization of Science and Technology, Ritsumeikan UniversityShiga, Japan; ^2^Molecular Neurobiology and Pharmacology, Graduate School of Medicine, The University of TokyoTokyo, Japan

**Keywords:** glutamate receptor δ2, motor learning, neurexin, parallel fiber, Purkinje cell, synapse formation

## Abstract

The cerebellum receives two excitatory afferents, the climbing fiber (CF) and the mossy fiber-parallel fiber (PF) pathway, both converging onto Purkinje cells (PCs) that are the sole neurons sending outputs from the cerebellar cortex. Glutamate receptor δ2 (GluRδ2) is expressed selectively in cerebellar PCs and localized exclusively at the PF-PC synapses. We found that a significant number of PC spines lack synaptic contacts with PF terminals and some of residual PF-PC synapses show mismatching between pre- and postsynaptic specializations in conventional and conditional GluRδ2 knockout mice. Studies with mutant mice revealed that in addition to PF-PC synapse formation, GluRδ2 is essential for synaptic plasticity, motor learning, and the restriction of CF territory. GluRδ2 regulates synapse formation through the amino-terminal domain, while the control of synaptic plasticity, motor learning, and CF territory is mediated through the carboxyl-terminal domain. Thus, GluRδ2 is the molecule that bridges synapse formation and motor learning. We found that the *trans*-synaptic interaction of postsynaptic GluRδ2 and presynaptic neurexins (NRXNs) through cerebellin 1 (Cbln1) mediates PF-PC synapse formation. The synaptogenic triad is composed of one molecule of tetrameric GluRδ2, two molecules of hexameric Cbln1 and four molecules of monomeric NRXN. Thus, GluRδ2 triggers synapse formation by clustering four NRXNs. These findings provide a molecular insight into the mechanism of synapse formation in the brain.

## Introduction

The cerebellum receives two excitatory afferents, the climbing fiber (CF) and the mossy fiber-parallel fiber (PF) pathway, both converging onto Purkinje cells (PCs) that are the sole neurons sending outputs from the cerebellar cortex. Glutamate receptors (GluRs) play central roles in synaptic transmission, synaptic plasticity, learning, memory, and development in the brain. Ionotropic GluRs have been classified into three major subtypes, the α-amino-3-hydroxy-5-methyl-4-isozaxole propionic acid (AMPA), kainate and *N*-methyl-D-aspartate (NMDA) receptors, based on the pharmacological, and electrophysiological properties (Mayer and Westbrook, 1987; Monaghan et al., 1989). We found the δ subtype of GluR by molecular cloning (Yamazaki et al., 1992). With respect to the amino-acid sequence identity, the GluRδ (GluD) subtype is positioned between the NMDA and non-NMDA (AMPA/kainite) subtypes (Yamazaki et al., 1992; Araki et al., 1993; Lomeli et al., 1993; Hollmann and Heinemann, 1994; Mori and Mishina, 1995; Mishina, 2000). GluRδ2, the second member of this subfamily, is selectively expressed in cerebellar PCs (Araki et al., 1993; Lomeli et al., 1993). Interestingly, GluRδ2 is localized at PF-PC synapses in cerebellar PCs, but not at CF-PC synapses (Takayama et al., 1996; Landsend et al., 1997). GluRδ2 knockout mice showed severe impairments of long-term depression (LTD) at the PF-PC synapse, motor learning, and motor coordination (Funabiki et al., 1995; Hirano et al., 1995; Kashiwabuchi et al., 1995; Kishimoto et al., 2001). Furthermore, a significant number of PC spines lack synaptic contacts with PF terminals and multiple CF innervation to PCs is sustained in GluRδ2 mutant mice (Kashiwabuchi et al., 1995; Kurihara et al., 1997; Hashimoto et al., 2001; Ichikawa et al., 2002). Thus, GluRδ2 plays a central role in the synaptic plasticity, motor learning, and neural wiring of cerebellar PCs. Since there is no evidence for GluRδ2 channel activities, although lurcher mutation (Ala639Thr) transformed GluRδ2 to constitutively active channels (Zuo et al., 1997), it remained unknown how GluRδ2 regulates cerebellar wiring and function. Recent findings provided significant insights on the issue.

## GluRδ2 regulates synaptic plasticity and motor learning through the C-terminal domain

**Figure 1:**
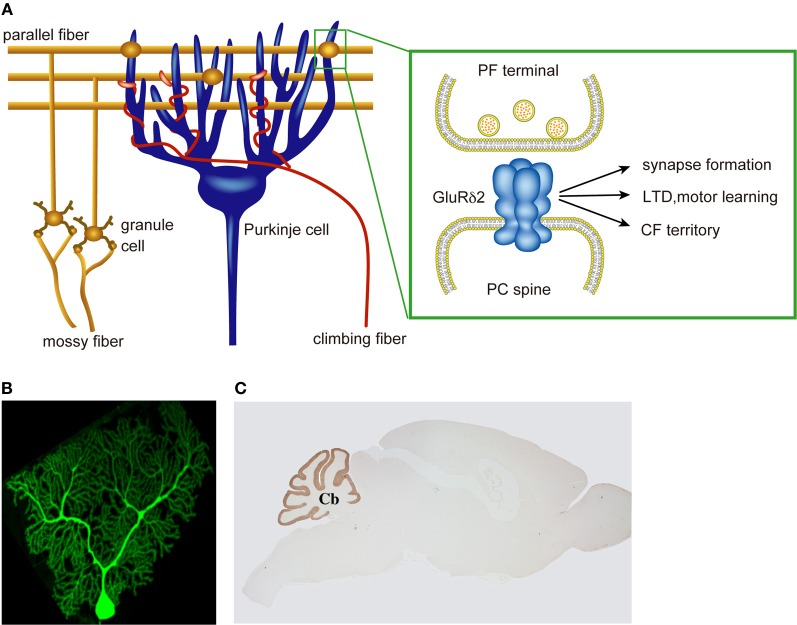
**Multiple roles of PC-specific GluR δ2 in cerebellar wiring and function. (A)** A cerebellar PC receives a large numbers of PF innervation at distal dendrites and a single CF innervation at proximal dendrites. GluRδ2 is selectively expressed in cerebellar PCs and exclusively localized at the PF-PC synapse. GluRδ2 at the PF synapse regulates PF-PC synapse formation, LTD induction, motor learning, and CF territory. **(B)** A cerebellar PC visualizes by EGFP. **(C)** Immunohistochemical staining of a mouse brain with anti-GluRδ2 antibody.

Studies with conventional and conditional knockout mice revealed that GluRδ2 is essential for synapse formation, synaptic plasticity, motor learning, and the restriction of CF territory (Figure 1). However, the causal relationships of these phenotypes remained to be clarified. The C-terminal cytoplasmic region of GluRδ2 contains at least three domains for protein-protein interactions (Roche et al., 1999; Uemura et al., 2004; Yawata et al., 2006). The postsynaptic density (PSD)-95/Discs large/zona occludens 1 (PDZ)-binding domain at the C-terminal, designated as the T site (Uemura et al., 2007), interacts with PSD-93, PTPMEG, Delphilin, nPIST, and S-SCAM (Roche et al., 1999; Hironaka et al., 2000; Miyagi et al., 2002; Yue et al., 2002; Yap et al., 2003). In the middle of the C-terminal cytoplasmic region, there is the domain that interacts with Shank scaffold proteins, designated as the S segment (Uemura et al., 2004). The membrane-proximal domain of the C-terminal cytoplasmic region of GluRδ2 interacts with PICK1 (Yawata et al., 2006).

We generated GluRδ2ΔT mice carrying mutant GluRδ2 lacking the T site comprising seven amino acids at the C-terminal (Uemura et al., 2007). There were no significant differences in the amount of receptor proteins in the PSD fraction and in the density of GluRδ2 immunogold particles at PF-PC synapses between wild-type and GluRδ2ΔT mice. Thus, the C-terminal truncation exerted little effect on the synaptic localization of receptor proteins. Synaptic connections between PF terminals and PC spines were intact in GluRδ2ΔT mice. However, LTD induction at PF-PC synapses was impaired and the improvement of the performance in the accelerating rotarod test was diminished in the mutant mice. The importance of the GluRδ2 C-terminal in cerebellar LTD and motor learning is consistent with the observations that in PTPMEG mutant mice, LTD at PF-PC synapses was significantly attenuated and rapid acquisition of the cerebellum-dependent delay eyeblink conditioning was impaired (Kina et al., 2007). These results suggest that the C-terminal T site of GluRδ2 is essential for LTD induction and motor learning, but is dispensable for PF-PC synapse formation (Uemura et al., 2007).

Delphilin is selectively expressed in cerebellar PCs except for a slight expression in the thalamus and is exclusively localized at the postsynaptic junction site of the PF-PC synapse (Miyagi et al., 2002). The characteristic expression pattern of Delphilin is reminiscent of GluRδ2. Delphilin knockout mice showed no detectable abnormalities in cerebellar histology, PC cytology, and PC synapse formation (Takeuchi et al., 2008). Delphilin ablation exerted little effect on the synaptic localization of GluRδ2. However, LTD induction was facilitated at PF-PC synapses and intracellular Ca^2+^ required for the induction of LTD appeared to be reduced in Delphilin knockout mice. We further showed that the gain-increase adaptation of the optokinetic response (OKR) was enhanced in the mutant mice. These findings suggest that synaptic plasticity at PF-PC synapses is a crucial rate-limiting step in OKR gain-increase adaptation, a simple form of motor learning (Takeuchi et al., 2008).

## GluRδ2 triggers PF-PC synapse formation by *trans*-synaptic interaction with neurexins through Cbln1

**Figure 2:**
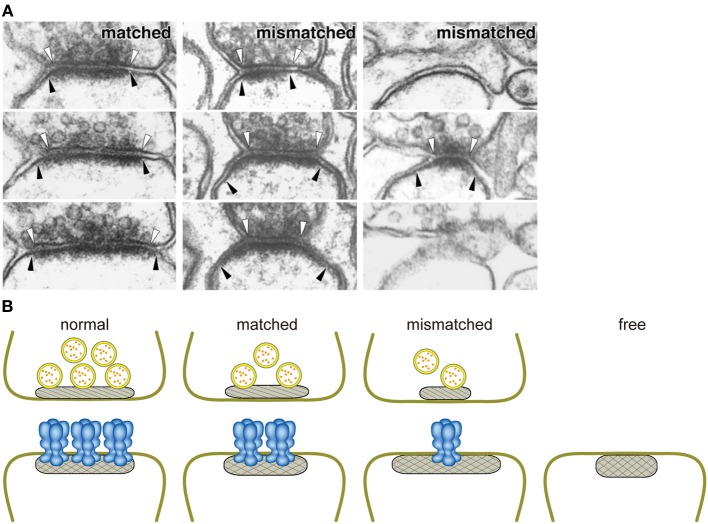
**Close relationship between the amount of GluRδ2 protein and the size of the active zone. (A)** Ablation of GluRδ2, when induced in the adult brain, resulted in the disruption of synaptic connections with PF terminals in a significant number of PC spines. In addition, some of residual PF-PC synapses show mismatching between pre- and postsynaptic specializations (Takeuchi et al., 2005). White and black arrowheads indicate the edges of active zone and PSD, respectively. **(B)** Schematic presentation of the relationships between the amount of GluRδ2 protein and the sizes of presynaptic active zone (hatched) and PSD (cross-hatched). The length of active zone became shorter in the order of normal, matched, and mismatched synapses according to the decrease of the density of GluRδ2-immunogold labeling at postsynaptic sites (Takeuchi et al., 2005). Based on the direct relationship between the density of postsynaptic GluRδ2 and the size of presynaptic active zones in GluRδ2 mutant mice, we proposed that GluRδ2 makes a physical linkage between the active zone and PSD by interaction with an active zone component. Normal, normal synapse of wild-type mice; matched, matched synapse of induced GluRδ2 KO mice; mismatched, mismatched synapse of induced GluRδ2 KO mice; free, free spine of induced GluRδ2 KO mice.

We examined the role of GluRδ2 in the adult brain using inducible and cerebellar PC-specific gene targeting on the C57BL/6 genetic background (Takeuchi et al., 2005). When GluRδ2 proteins were diminished, a significant number of PC spines lost their synaptic contacts with PF terminals. Thus, studies with conventional and inducible knockout mice indicate that the formation and maintenance of PF-PC synapses are critically dependent on GluRδ2 *in vivo* (Kashiwabuchi et al., 1995; Takeuchi et al., 2005). Concomitant with the decrease of postsynaptic GluRδ2 proteins, presynaptic active zones shrank progressively and PSD expanded, resulting in mismatching between pre- and postsynaptic specializations at PF-PC synapse (Figure 2). Furthermore, GluRδ2 and PSD-93 proteins were concentrated at the contacted portion of mismatched synapses, while AMPA receptors distributed in both the contacted and dissociated portions. Thus, postsynaptic GluRδ2 is a key regulator of the presynaptic active zone and PSD organization at PF-PC synapses. Based on the direct relationship between the density of postsynaptic GluRδ2 and the size of presynaptic active zones in GluRδ2 mutant mice generated by inducible Cre-mediated ablation, we proposed that GluRδ2 makes a physical linkage between the active zone and PSD by direct or indirect interaction with an active zone component (Takeuchi et al., 2005). Indirect interaction through PSD proteins appears to be less likely since the C-terminal truncation of GluRδ2 has little effect on PF-PC synapse formation, while the mutation impairs cerebellar LTD and motor learning (Uemura et al., 2007).

**Figure 3:**
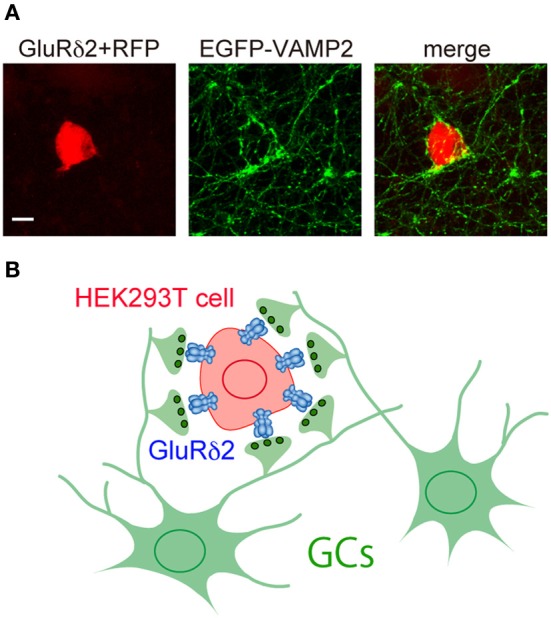
**Induction of presynaptic differentiation by GluR δ 2. (A)** HEK293T cells transfected with expression vectors for GluRδ2 and tagRFP (red) were seeded on top of cultured cerebellar neurons transfected with an expression vector for vesicle-associated membrane protein-2 (VAMP-2) fused with EGFP at its N terminus (EGFP-VAMP2) (green). After 2 days of co-culture, cells were immunostained for EGFP. Note that numerous EGFP-VAMP2 signals accumulated on the surface of HEK293T cells expressing GluRδ2. Scale bar represents 10 μm. **(B)** Schematic presentation of the accumulation of GC axon terminals on the surface of HEK293T cells expressing GluRδ2.

To identify the key domain responsible for synapse formation, we expressed GluRδ2 in HEK293T cells and cultured the transfected cells with cerebellar granule cells (GCs) (Uemura and Mishina, 2008) (Figure 3). Numerous punctate signals for presynaptic markers were observed on the surface of HEK293T cells expressing GluRδ2. The presynaptic specializations of cultured GCs induced by GluRδ2 were capable of exo- and endocytosis as indicated by FM1-43 dye labeling. Replacement of the extracellular N-terminal domain (NTD) of GluRδ2 with that of the AMPA receptor GluRα1 abolished the inducing activity. The NTD of GluRδ2 (GluRδ2-NTD) coated on beads successfully induced the accumulation of presynaptic specializations. These results suggest that GluRδ2 triggers synapse formation by direct interaction with presynaptic component(s) through the NTD (Uemura and Mishina, 2008; Kakegawa et al., 2009; Kuroyanagi et al., 2009; Mandolesi et al., 2009).

To seek for GluRδ2 interacting proteins, the presynaptic differentiation of cerebellar GCs was induced by treatment with GluRδ2-NTD-coated magnetic beads and then surface proteins of cerebellar GC axons were covalently bound to GluRδ2-NTD using non-permeable cross-linker 3,3′-dithiobis(sulfosuccinimidylpropionate). Comparative analysis of the isolated proteins by liquid chromatography-tandem mass spectrometry identified neurexin (NRXN) 1, NRXN2, FAT2, protein tyrosine phosphatase σ (PTPσ), and cerebellin 1 precursor protein (Cbln1) as possible GluRδ2-interacting proteins (Uemura et al., 2010). NRXN1, NRXN2, FAT2, and PTPσ are membrane proteins (Pulido et al., 1995; Nakayama et al., 2002; Südhof, 2008), while Cbln1 is a glycoprotein secreted from cerebellar GCs (Bao et al., 2005). After a series of selections, we found robust binding signals of GluRδ2-NTD on the surface of HEK293T cells transfected with NRXN1β or NRXN2β in the presence of Cbln1. It is known that presynaptic NRXNs bind to postsynaptic neuroligins (NLGNs) forming *trans*-synaptic cell adhesion complexes (Ichtchenko et al., 1995; Scheiffele et al., 2000; Graf et al., 2004) and NLGNs preferentially bind to NRXN variants lacking splice segment 4 (S4) (Boucard et al., 2005; Chih et al., 2005; Comoletti et al., 2006). In contrast to NLGNs, GluRδ2 selectively interacts with NRXN variants containing S4. NRXN variants containing S4 were expressed in the cerebellum but those lacking S4 were hardly detectable except for early stages of development, while both variants were found in the cerebral cortex and hippocampus (Uemura et al., 2010; Iijima et al., 2011).

**Figure 4:**
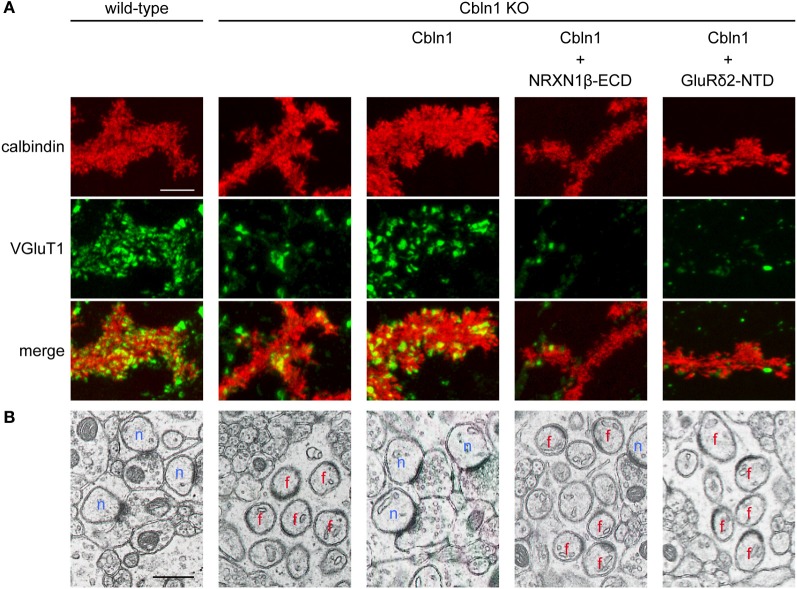
**The GluR δ2-Cbln1-NRXN *trans*-synaptic triad mediates synapse formation (modified from Uemura et al., 2010). (A)** Suppression of Cbln1 synaptogenic activity by the extracellular domain of NRXN1β (NRXN1β-ECD) and the N-terminal domain of GluRδ2 (GluRδ2-NTD) in cultured cerebellar neurons. In primary cultures of cerebellar neurons, numerous punctate staining signals for VGluT1 were found on the dendrites of PCs from wild-type mice. VGluT1 signals were significantly reduced in PCs from Cbln1 KO mice. Addition of Cbln1 restored the intensity of VGluT1 signals. The restoring activity of Cbln1 was suppressed by addition of NRXN1β-ECD and GluRδ2-NTD. **(B)** Suppression of Cbln1 synaptogenic activity by NRXN1β-ECD and GluRδ2-NTD *in vivo*. Electron micrographs of cerebella from wild-type and Cbln1 KO mice and those from Cbln1 KO mice injected with Cbln1 together with or without NRXN1β-ECD and GluRδ2-NTD. In wild-type mice, all PC spines formed synaptic contacts with PFs. In Cbln1 KO mice, many PC spines lacked synaptic contacts (free spines). Injection of Cbln1 restored PF-PC connections in Cbln1 KO mice. The *in vivo* synatogenic activity of Cbln1 was suppressed by co-injection of NRXN1β-ECD and GluRδ2-NTD. n, normal synapses; f, free spines. Scale bars represent 5 μm in **(A)** and 0.5 μm in **(B)**.

Direct binding experiments showed that GluRδ2 is a receptor for Cbln1 and NRXN is another receptor for Cbln1 (Uemura et al., 2010). The K_D_ value of Cbln1 for the NTD of GluRδ2 estimated by surface plasmon resonance binding assays is 16.5 nM and that for the extracellular domain (ECD) of NRXN1β is 0.17 nM. These values suggest high affinity interactions of GluRδ2, Cbln1 and NRXN as compared with K_D_ values (~200 to ~600 nM) reported for the interactions between NLGNs and NRXNs (Comoletti et al., 2003; Koehnke et al., 2008). Matsuda et al. (2010) also reported the interaction between Cbln1 and GluRδ2. Since Cbln1 is a ligand for both GluRδ2 and NRXN, we propose a model in which postsynaptic GluRδ2 interacts with presynaptic NRXN through Cbln1 and this ternary interaction provides a physical linkage between PSD and active zone (Uemura et al., 2010). The synaptogenic activity of GluRδ2 is hindered by knockout of Cbln1 and by small interference RNA-mediated knockdown of NRXNs. Furthermore, the synaptogenic activity of Cbln1 in cerebellar primary cultures and *in vivo* was abolished by the NTD of GluRδ2 and the ECD of NRXN1β (Figure 4). These results suggest that the *trans*-synaptic interaction of postsynaptic GluRδ2 and presynaptic NRXNs through Cbln1 mediates PF-PC synapse formation in the cerebellum (Uemura et al., 2010). This model well explains previous observations that the size of the presynaptic active zone shrank progressively concomitant with the decrease of postsynaptic GluRδ2 proteins upon inducible Cre-mediated GluRδ2 ablation (Takeuchi et al., 2005) and that Cbln1 knockout mice phenotypically mimic GluRδ2 knockout mice (Hirai et al., 2005).

## Assembly stoichiometry of the *trans*-synaptic triad

Cumulative evidence indicates the tetrameric assembly of the AMPA/kainate- and NMDA-type GluRs (Laube et al., 1998; Rosenmund et al., 1998; Bowie and Lange, 2002; Sun et al., 2002; Weston et al., 2006). The mobility of GluRδ2 molecules from the membrane fraction corresponded to the size of the tetramer in blue native PAGE. GluRδ2 band collapsed into monomeric and dimeric intermediates by the treatment of 1% SDS. These behaviors were similar between GluRδ2 and AMPA-type GluR. These results suggest that GluRδ2 exists as a tetramer in the membrane. On the other hand, GluRδ2-NTD assembled into a stable homodimer. The NTD of ionotropic GluRs with tetrameric structure assembles as a dimer of dimers (Schorge and Colquhoun, 2003; Tichelaar et al., 2004; Midgett and Madden, 2008; Kumar et al., 2009) and tetrameric iGluRs have 2-fold symmetry rather than 4-fold symmetry (Armstrong and Gouaux, 2000; Sobolevsky et al., 2004, 2009; Nanao et al., 2005).

When incubated with cultured cerebellar GCs, dimeric GluRδ2-NTD exerted little effect on the intensities of punctate immunostaining signals for Bassoon and vesicular glutamate transporter 1 (VGluT1). In contrast, tetrameric GluRδ2-NTD prepared by cross-linking dimeric GluRδ2-NTD-Fc using F(ab′)_2_ of anti-Fc antibody enhanced the accumulation of the active zone and synaptic vesicle proteins in axons of cultured GCs. These results suggest that native GluRδ2 is assembled into a tetramer and this tetrameric assembly is essential for GluRδ2 to induce presynaptic differentiation (Lee et al., 2012).

Affinities of a series of Cbln1 mutants for GluRδ2-NTD and NRXN1β-ECD suggest that the binding sites of Cbln1 for GluRδ2 and NRXN1β are differential rather than identical. In addition, no competition was detectable in the binding to Cbln1 between GluRδ2-NTD and the laminin–neurexin–sex hormone-binding globulin (LNS) domain of NRXN1β during triad formation. These results suggest that GluRδ2 and Cbln1 interact with each other rather independently of Cbln1-NRXN1β interaction and vice versa. We thus examined the assembly stoichiometries of GluRδ2-Cbln1 and Cbln1-NRXN1β complexes one by one. Both fast protein liquid chromatography gel-filtration assay and isothermal titration calorimetry analysis consistently showed that dimeric GluRδ2-NTD and hexameric Cbln1 assembled in the molar ratio of one to one, while hexameric Cbln1 and monomeric NRXN1β-LNS assembled in the molar ratio of one to two. Since native GluRδ2 exists as a tetramer in the membrane and the tetramerization is essential for GluRδ2-NTD to stimulate the accumulation of Bassoon and VGluT1 in the axons of cultured GCs, we suggest that the synaptogenic triad is composed of one molecule of tetrameric GluRδ2, two molecules of hexameric Cbln1 and four molecules of monomeric NRXN (Lee et al., 2012).

## Mechanism of GluRδ2-mediated synapse formation

**Figure 5:**
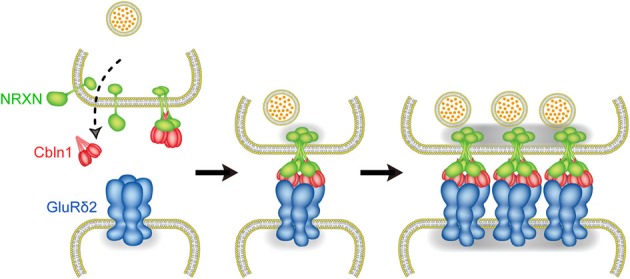
**Molecular mechanism of PF-PC synapse formation.** Before PF-PC synapse formation, Cbln1 secreted from cerebellar GCs may interact with presynaptic NRXNs. Cbln1-induced NRXN dimerization is not sufficient to trigger presynaptic differentiation. When the contact between the PF terminal and PC spine takes place, GluRδ2 triggers synapse formation by clustering four NRXNs through triad formation.

During development, axons of immature neurons show a capacity for evoked recycling of synaptic vesicles and clusters of the vesicles along axonal segments, even in the absence of target cells (Ziv and Garner, 2004; Jin and Garner, 2008). However, the synaptic vesicle aggregation, in the absence of a postsynaptic contact, is not stably anchored to a given region of the cell surface. Contacts with postsynaptic sites trigger the stabilization and maturation of synapses. In cultured cerebellar GCs, the majority of varicosities containing presynaptic proteins are not apposed to definite postsynaptic structures (Marxen et al., 1999; Urakubo et al., 2003). Cbln1 is a high-affinity ligand for NRXNs (Uemura et al., 2010; Joo et al., 2011) and is secreted from cerebellar GCs (Bao et al., 2005), suggesting that the interaction between secreted Cbln1 and presynaptic NRXNs takes place before PF-PC synapse formation. However, punctate staining signals for Bassoon were comparable between GC cultures from wild-type and Cbln1 knockout mice. The addition of Cbln1 to GC cultures exerted little effect on the intensity of Bassoon signals. Thus, the formation of NRXN dimers is not sufficient to induce presynaptic differentiation. Consistently, GluRδ2-NTD dimer that binds to one molecule of Cbln1 failed to induce presynaptic differentiation. In contrast, GluRδ2-NTD tetramer stimulated the accumulation of punctate signals for active zone protein Bassoon and synaptic vesicle protein VGluT1 in cultured cerebellar GCs. Since GluRδ2-NTD tetramer is soluble, it is unlikely that this stimulating effect is due to anchoring presynaptic proteins. Our results suggest that tetrameric GluRδ2-NTD assembles two molecules of Cbln1 and four molecules of NRXNs, whereas dimeric GluRδ2-NTD interacts with one molecule of Cbln1 and two molecules of NRXNs. Thus, clustering of four NRXNs by tetrameric GluRδ2-NTD via two Cbln1 is a key step to trigger presynaptic differentiation (Lee et al., 2012). Taken together, our results suggest the mechanism of PF-PC synapse formation as follows. Cbln1 secreted from cerebellar GCs may interact with presynaptic NRXNs before PF-PC synapse formation. However, Cbln1-induced NRXN dimerization is not sufficient to trigger presynaptic differentiation. When the contact between the PF terminal and PC spine takes place, GluRδ2 triggers synapse formation by clustering four NRXNs through triad formation (Figure 5). Since NRXNs interact with synaptotagmin, CASK, Mint and syntenin through its C-terminal (Hata et al., 1993, 1996; Butz et al., 1998; Biederer and Südhof, 2000; Grootjans et al., 2000) and the C-terminal of NRXN is critical for the induction of presynaptic differentiation *in vitro* (Dean et al., 2003), tetramerization of NRXNs may stimulate the clustering of these scaffold proteins leading to the organization of transmitter release machineries (Butz et al., 1998; Maximov et al., 1999; Biederer and Südhof, 2000, 2001).

## Conclusion

Cerebellar PC-specific GluRδ2 plays essential roles in synapse formation, synaptic plasticity and motor learning. The NTD of GluRδ2 is responsible for synapse formation, whereas the C-terminal domain is essential for LTD induction and motor learning. Thus, GluRδ2 is the molecule that bridges synapse formation and motor learning in the cerebellum.

Synapse formation is the key step in the development of neuronal networks. Precise synaptic connections between nerve cells in the brain provide the basis of perception, learning, memory, and cognition. Although a number of *trans*-synaptic cell adhesion molecules have been identified that play roles in pre- and postsynaptic differentiation of cultured hippocampal neurons, the precise roles of these molecules in synapse formation *in vivo* remain elusive (Scheiffele et al., 2000; Dean et al., 2003; Graf et al., 2004; Waites et al., 2005; Varoqueaux et al., 2006; Dalva et al., 2007; McAllister, 2007; Südhof, 2008; Shen and Scheiffele, 2010; Williams et al., 2010; Siddiqui and Craig, 2011). Our results provide evidence that the *trans*-synaptic interaction of postsynaptic GluRδ2 and presynaptic NRXNs through Cbln1 mediates PF-PC synapse formation *in vivo* in the cerebellum (Uemura et al., 2010). Furthermore, the stoichiometry of synaptogenic GluRδ2-Cbln1-NRXN triad suggests that GluRδ2 triggers presynsptic differentiation by clustering four NRXNs (Lee et al., 2012). It will be essential for the elucidation of synaptogenesis mechanism to investigate how NRXN clustering initiates the formation of presynaptic active zone. Interestingly, approximately half of PF-PC synapses survived in GluRδ2 knockout mice (Kashiwabuchi et al., 1995; Kurihara et al., 1997). There may be at least two types of PF-PC synapses, GluRδ2-dependent and independent synapses. Alternatively, other synaptogenic molecule(s) may partly compensate GluRδ2 deficiency in the knockout mice. It should be noted that the organization and composition of remaining PF-PC synapses in the absence of GluRδ2 appear to be altered, suggesting that GluRδ2 also plays a role as a PSD organizer (Takeuchi et al., 2005; Yamasaki et al., 2011). Further investigation of the structure and function of the GluRδ2-Cbln1-NRXN synaptogenic triad will provide a clue to understand how central synapses are formed, mature, show plastic changes, and mediate learning and memory.

During development, PC circuitry is established through heterosynaptic competition between PFs and CFs (Mariani et al., 1977; Crépel, 1982). GluRδ2 regulates the PC wiring by suppressing invasion of CF branches to the territory of PF innervation and to neighboring PCs (Kashiwabuchi et al., 1995; Hashimoto et al., 2001; Ichikawa et al., 2002; Uemura et al., 2007; Miyazaki et al., 2010). Weakened PF inputs due to the decrease of PF-PC synapses in GluRδ2 mutant mice may result in CF invasion to the PF territory (Hashimoto et al., 2001; Ichikawa et al., 2002). However, the territory of CF innervation expanded distally to spiny branchlets in GluRδ2ΔT mice with intact PF-PC synaptic connections (Uemura et al., 2007). GluRδ2 is localized at PF-PC synapses but not at CF synapses (Takayama et al., 1996; Landsend et al., 1997). Thus, GluRδ2 should suppress the distal extension and ectopic innervation of CF axon terminals by the signaling through the C-terminal T site (Uemura et al., 2007).

### Conflict of interest statement

The authors declare that the research was conducted in the absence of any commercial or financial relationships that could be construed as a potential conflict of interest.
